# Radiofrequency Wave Sensing for Rapid Animal Health Monitoring: A Proof-of-Concept Study

**DOI:** 10.3390/vetsci12111096

**Published:** 2025-11-18

**Authors:** Aftab Siddique, Ramya Kota, Goutham Kumar Isai, Davia Brown, Oreta Samples, Niki Whitley, Phaneendra Batchu, Thomas H. Terrill, Jan van Wyk

**Affiliations:** 1 Department of Agricultural Sciences, Fort Valley State University, Fort Valley, GA 31030, USA; rkota@wildcat.fvsu.edu (R.K.); gisai@wildcat.fvsu.edu (G.K.I.); dbrow212@wildcat.fvsu.edu (D.B.); sampleso@fvsu.edu (O.S.); whitleyn@fvsu.edu (N.W.); terrillt@fvsu.edu (T.H.T.); 2 Department of Veterinary Tropical Diseases, Faculty of Veterinary Science, University of Pretoria, Private Bag x04, Onderstepoort 0110, South Africa; jan.vanwyk@up.ac.za

**Keywords:** classification model, FAMACHA©, livestock, multilayer perceptron, parasitism, predictive modeling, radio-frequency waves, small ruminants, *Haemonchus*

## Abstract

Anemia caused by gastrointestinal parasitism is a major constraint to small ruminant productivity, particularly in low-resource production systems where diagnostic tools and veterinary access are limited, with use of FAMACHA as a biological reference This study evaluated the potential of radio-frequency non-destructive technique (RF-NDT) wave-derived features as non-invasive biomarkers for anemia detection in goats, using FAMACHA© scores as a biological reference. Variable clustering of the top ten frequencies revealed distinct patterns across health states. Healthy (FAMACHA© 1) animals were characterized by a single frequency cluster centered at 8.43 GHz, which explained 93.7% of variation, whereas moderately affected animals (FAMACHA© 2) shifted to 9.33 GHz with reduced uniformity (88.7%). Borderline animals (FAMACHA© 3) required two clusters (9.89 and 8.23 GHz), explaining 91.0% of variation, indicating increasing tissue heterogeneity with anemia progression. Regression analysis demonstrated strong predictive power, with Linear Regression achieving R^2^ = 1.00 and Random Forest R^2^ = 0.79 (RMSE = 0.07), Support Vector Regression underperformed (R^2^ = 0.31). Classification models confirmed the feasibility of categorical anemia detection. The Multilayer Perceptron achieved the highest accuracy (0.84), F1-score (0.83), and ROC-AUC (0.94), outperforming Support Vector Machine (accuracy 0.67, F1 = 0.67) and K-Nearest Neighbors (accuracy 0.60, F1 = 0.61). These findings establish proof-of-concept that RF waves capture physiologically meaningful dielectric signatures linked to anemia, reflecting hemoglobin concentration, hydration, and microcirculatory function. The integration of RF sensing with machine learning offers a rapid, and non-invasive scalable diagnostic approach. Future work should expand validation across breeds and environments, optimize sensor design, and embed neural classifiers for field-ready deployment.

## Simple Summary

In farming systems with limited veterinary access, parasitic anemia in goats and other small ruminants is a major issue. Farmers typically use manual FAMACHA© scoring, which is time-consuming and somewhat lacking in consistency. The use of radio-frequency (RF) waves like those used in wireless communication were investigated to detect goat anemia quickly and non-invasively. Clear animal health patterns emerged from researching how different frequencies interact with the organism. Healthy goats responded uniformly at 8.43 GHz, but anemic goats switched at 9.33 GHz. Anemia complicates the body’s reaction to RF waves, since borderline anemic goats needed two frequencies (9.89 and 8.23 GHz) to explain the variation. It was also examined if machine learning models might predict animal health using RF waves. The linear regression model accurately predicted FAMACHA© scores, but the Random Forest model was more resilient to biological variation. The most accurate neural network model for classification was Multilayer Perceptron, which identified anemia types with 84% accuracy and a 94% ROC-AUC. Our findings demonstrate that RF wave detection and artificial intelligence can monitor goat health quickly, reliably, and non-invasively. This method could help farmers control parasites and enhance animal welfare by replacing manual scoring.

## 1. Introduction

Small ruminants play a critical role in sustainable livestock production systems globally due to their capacity to thrive in marginal environments and provide essential resources [1]. Goat products, including high-quality protein, milk, and fiber, contribute significantly to the livelihoods of millions of households, particularly in developing regions where other livestock may struggle to survive [2]. However, despite these advantages, small ruminants face numerous health challenges that impede their productivity, such as parasitism, metabolic stress, and subclinical diseases. These health issues lead to considerable economic losses and jeopardize the sustainability of production systems that depend on these animals [3].

The economic impact of health challenges, particularly due to parasitic infections, is significant [4]. For instance, gastrointestinal nematodes, predominantly *Haemonchus contortus*, have been identified as a leading cause of morbidity and mortality in sheep and goats. Effective management of these infections is further complicated by the growing resistance to commonly used anthelmintics, which has been thoroughly documented in numerous studies [5,6]. This situation underscores the necessity for continuous monitoring and effective control strategies to alleviate the burden of parasitism in small ruminants.

Traditional monitoring practices, such as body condition scoring, FAMACHA© charting, and periodic Packed Cell Volume (PCV) hematological testing, have served as essential tools for assessing the health status of these animals [7]. Yet, they also present several shortcomings such as primarily being labor-intensive, subjective for addressing health issues.

The FAMACHA© system has been recognized for its ability to identify anemia due to *H. contortus* infections with high sensitivity as to *H. contortus* infection [8]. However, its performance can vary significantly depending on the training of the personnel conducting the assessments, which can lead to discrepancies and render results somewhat subjective [9]. Additionally, reliance on visual assessments can introduce inconsistencies in health monitoring, making it imperative to seek more robust and precise alternatives. The FAMACHA© chart remains a practical tool for detecting anemia; however, its accuracy depends on the user’s training, experience, and ambient lighting conditions. In this study, a radio-frequency (RF) sensing approach was introduced to generate numerical dielectric measurements reflective of tissue composition and hydration status. This method enables objective, reproducible, and user-independent evaluation of physiological condition. By transitioning from observer-based scoring to data-driven sensing, the system directly mitigates the subjectivity inherent in manual assessment.

As the demand for efficient, welfare-oriented, and climate-resilient livestock production systems increases, there is an emergent need for innovative approaches to animal health monitoring. For example, more sophisticated analytics such as optimizing deductions via Artificial Intelligence for optimal early detection of especially relatively slowly declining animal health conditions, including hemonchosis, under less-than-optimal epidemiological conditions. One promising direction lies in the integration of non-invasive and scalable technologies that can provide real-time health assessments. For example, advances in machine learning applications for predicting anthelmintic resistance and managing gastrointestinal nematode infections show potential for transforming traditional practices into more automated and precise methodologies [10]. The use of technology, such as digital applications that can analyze data collected from various health indicators (e.g., blood packed cell volume (PCV), fecal egg counts or physical health assessments), including such as the following, which could streamline health monitoring, allowing for timely interventions when health declines are detected [11].

### Radio-Wave Frequency

Radio-frequency (RF) wave sensing represents a promising technology in this regard. The RF waves, occupying the electromagnetic spectrum between 2 GHz and 18 GHz, have unique penetration and interaction properties with biological tissues [12]. When RF waves pass through or reflect from biological materials, they generate distinctive signatures determined by tissue composition, density, hydration status, and structural properties [13]. These dielectric properties make RF waves particularly attractive for non-destructive and non-invasive sensing applications. Unlike optical approaches, which are limited to surface-level features, RF signals can penetrate tissues, enabling the detection of subsurface physiological changes [14,15]. Moreover, RF systems can operate with minimal sample preparation, offer rapid measurement times, and are adaptable to field conditions, making them well-suited for livestock applications.

The concept of RF-based monitoring has been explored in several agricultural contexts. In cereals and grains, RF spectroscopy has been used to estimate moisture content and bulk density with high accuracy, supporting postharvest management [16]. Similarly, in poultry science, RF and microwave sensors have been evaluated as alternatives to near-infrared and hyperspectral imaging for detecting muscle myopathies, such as woody breast, due to their ability to capture tissue-level differences in water content and structure [15]. Translating this principle to small ruminants, RF sensing has the potential to detect early physiological alterations associated with health and welfare conditions such as anemia, hydration deficits, and changes in muscle or fat composition before they are clinically observable [17]. Thus, the application of RF-based methods could provide producers with a rapid, non-invasive, and scalable system for monitoring animal health in real time.

The rationale for pursuing alternative non-destructive RF (RF-NDT) wave sensing in small ruminant health monitoring is threefold. First, RF technology offers objectivity and repeatability, overcoming the biases and inconsistencies of manual scoring systems. Second, it provides a non-invasive and rapid diagnostic approach, reducing the need for invasive sampling or repeated handling that stresses animals and requires skilled labor. Third, RF sensing aligns with sustainability and welfare goals, as it enables early detection and targeted interventions, thereby reducing mortality, lowering drug use, and improving feed efficiency.

Despite these advantages, gaps in knowledge remain. The application of RF wave sensing in live small ruminants has not been extensively studied, and its ability to discriminate specific health-related physiological changes requires empirical validation. Biological variability across breeds, ages, and production environments introduces challenges for developing robust predictive models [18,19,20]. Furthermore, while RF waves are theoretically well-suited for tissue characterization, their utility in differentiating subtle variations associated with parasitism or metabolic status in live animals has yet to be systematically investigated. These uncertainties highlight the need for proof-of-concept studies to establish baseline evidence for feasibility and to guide future development of integrated animal health monitoring systems.

The present study addresses this gap by evaluating the feasibility of using a RF wave device operating between 2 and 18 GHz as a non-destructive proof-of-concept tool for small ruminant health monitoring. Specifically, this study investigates whether RF wave interactions with biological tissues can provide measurable signatures that may serve as proxies for physiological status. By generating and analyzing RF-based datasets, the goal is to assess the potential of this approach as a foundation for future multimodal sensing platforms. While the present work is exploratory, it is envisioned as a steppingstone toward the development of comprehensive animal health monitoring systems that integrate RF sensing with complementary modalities and AI-driven analytics to enhance precision, sustainability, and resilience in small ruminant production.

## 2. Materials and Methods

### 2.1. Animals and Housing

A total of eight intact male Spanish goats (*Capra hircus*) were used in this proof-of-concept study. The animals were 24 months of age and weighed between 36 and 50 kg at the time of data collection. All goats were maintained at the Fort Valley State University (FVSU) Agriculture Technology Center farm in Fort Valley, Georgia, USA. The animals were raised under standard husbandry conditions, with unrestricted access to fresh water and grazing pastures. Goats were managed on a mixed-grass pasture system that permitted natural exposure to gastrointestinal nematodes during the grazing season (March–September 2023). This natural grazing regime ensured that the animals developed physiological variability in parasitic status and hematological indicators (e.g., PCV), under field conditions while allowing for the evaluation of RF sensing under realistic management scenarios.

### 2.2. Experimental Design

The proof-of-concept study employed a longitudinal, non-invasive design in which RF measurements were repeatedly collected from each goat at multiple body locations. Although only eight goats were used, the design leveraged repeated within-animal sampling to capture intra- and inter-individual variation in RF signatures over time. Four anatomical sides per goat (left lateral, right lateral, dorsal, and ventral regions) were selected for RF data acquisition, thereby maximizing spatial coverage of body tissues and physiological states. This approach yielded a robust dataset while minimizing animal numbers, in line with the 3Rs principle (Replacement, Reduction, and Refinement) of ethical animal use.

### 2.3. Device Safety and Certification

The RF sensor unit functions at low power and possesses C1D2 (Class 1 Division 2) certification, guaranteeing safe operation in environments potentially containing combustible gasses or vapors under abnormal conditions. The certification verifies that the device design inhibits sparking, overheating, or igniting, in accordance with safety requirements. This study utilized equipment in conventional animal housing and laboratory environments, devoid of such dangers. The C1D2 certification offers enhanced assurance of electrical safety and non-incendive functionality, hence facilitating its applicability in many agricultural and healthcare fields.

### 2.4. Radio-Frequency Wave Data Collection

To confirm the physiological validity of the FAMACHA© scoring system, packed cell volume (PCV) was measured using the standard microhematocrit centrifugation method. The mean ± standard deviation of PCV for each FAMACHA© category was 30.74 ± 2.23% (Score 1), 24.99 ± 1.48% (Score 2), and 20.43 ± 1.32% (Score 3) [21,22]. The progressive decline in PCV with increasing FAMACHA© scores demonstrated that the field-based classifications accurately reflected the severity of hematological anemia. In this proof-of-concept, FAMACHA© served only as a pragmatic field label; RF is intended as a non-invasive screening adjunct to prioritize animals for confirmatory PCV and/or fecal egg counts, not a replacement.

Radio-frequency wave signals were collected using a custom-designed RF device (Compass technology group, LLC, Alpharetta, GA, USA) consisting of a broadband transmitting–receiving antenna pair and an integrated vector network analyzer module, enabling dielectric reflectometry configured to emit and record signals across a frequency range of 2 to 18 GHz. The device performed a continuous frequency sweep across the 2–18 GHz spectrum, consisting of 1601 evenly spaced frequency steps per scan (Supplementary File, Figure S1). This configuration enabled high-resolution spectral acquisition suitable for identifying narrowband dielectric changes in biological tissue. From these spectra, the top ten discriminative frequencies (8.23, 8.43, 8.67, 8.89, 9.11, 9.33, 9.56, 9.78, 9.89, and 10.12 GHz) were selected based on false discovery rate (FDR) filtering and Bootstrap Forest screening for downstream modeling.

**Figure 1:**
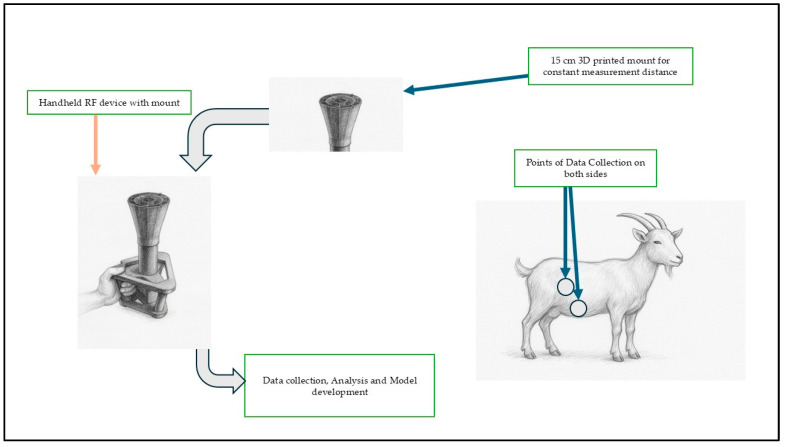
Experimental setup showing the handheld RF device with a 15 cm 3D-printed mount, measurement points on both lateral sides of the goat, and workflow for RF data collection and model development.

Animals were restrained in a standing posture, and data were collected from left and right ventral regions, and under the stomach area on both sides. Each scan lasted 5–10 s and produced 1601 discrete frequencies on each of the readings across the band, resulting in 6404 data points per animal per session. This spectrum was selected because it spans both ultra-high frequency (UHF) and super-high frequency (SHF) bands, enabling sufficient penetration and interaction with soft tissues, while preserving sensitivity to dielectric properties of biological material. For each anatomical side, a total of 1601 discrete frequencies were scanned. Across all eight goats, the experiment produced a cumulative dataset of 51,232 raw RF observations. Measurements were conducted with the animals in a standing, restrained position to minimize movement noises, and the device was held at a standardized distance (15 cm) and orientation relative to the body surface to ensure measurement consistency (Figure 1).

### 2.5. Data Preprocessing

Analysis followed a seven-step pipeline: (i) clean and normalize raw amplitude/phase spectra (Supplementary File, Figure S2); (ii) apply FDR across the full 2–18 GHz bands (Supplementary File, Figure S3); (iii) run Bootstrap-Forest predictor screening (Supplementary File, Figure S4); (iv) select the top 10 discriminative frequencies (Supplementary File, Figure S5); (v) perform variable clustering; (vi) balance classes with SMOTE [23]; and (vii) train and evaluate models with nested 10-fold cross-validation [24,25].

After acquisition, RF data were exported for preprocessing and modeling. Raw frequency-domain readings were cleaned to remove noise from the environment, and prevent motion, or instrument drift, before being filtered, normalized, and smoothed in the frequency domain. The cleaned data were prepared for machine learning, in order to allow frequency signatures to be assessing as predictors of physiological or health states. Although this is a proof-of-concept study, the strict adherence to best practices for RF signal analysis reassures the reliability of our results and supports reproducibility and future integration into predictive analytics pipelines, consistent with Siddique et al. [15].

### 2.6. Ethical Considerations

All animal use protocols were reviewed and approved by the Fort Valley State University Agricultural and Laboratory Animal Care and Use Committee (ALACUC approval number F-T-01-2022). The study was designed to comply fully with institutional and national regulations governing animal welfare. In particular, care was taken to reduce conditions that could lead to mortality or distress in the experimental goats, especially those associated with low PCV values caused by parasitic infection. No animals were sacrificed for this study, and all goats were monitored daily by trained personnel for health and welfare throughout the experimental period. The study adhered to the guiding principles of minimizing animal numbers, refining procedures to reduce stress, and replacing invasive techniques with non-invasive monitoring approaches whenever feasible.

## 3. Data Analysis

The raw RF data collected from the device, consisting of both amplitude and phase components across the 2–18 GHz frequency range, were exported for computational processing. Data preprocessing and statistical analyses were conducted using Python (Ver. 3.10) and JMP Pro 16.0 (SAS Institute Inc., Cary, NC, USA). The analytical workflow was designed to systematically clean, screen, and extract meaningful frequency-domain features associated with physiological variation, while minimizing the risk of spurious findings inherent with high-dimensional datasets [15].

For consistency across models and a common plotting scale, we treated the ordinal labels (1–3) as evenly spaced and applied a min-max mapping to preserve order and spacing while standardizing the target range for regression and visualization: 1 → 0.0, 2 → 0.5, 3 → 1.0 [15]. Initial data screening was performed to identify and remove noise that could arise from environmental interference, device variability, or motion during measurement. To reduce false positives and control for Type I error rates, a false discovery rate (FDR) analysis was applied to the complete RF spectrum [15]. This approach allowed us to filter out spurious associations and retain only frequencies with a high likelihood of biological relevance. Subsequently, the Predictor Screening (PS) module in JMP Pro was employed, using a Bootstrap Forest algorithm, to isolate frequency ranges most strongly associated with classification outcomes [23]. The combination of FDR and Bootstrap Forest screening ensured that the retained predictors were statistically robust and biologically interpretable.

From this two-step screening process, the top ten discriminative frequencies were selected for further analysis. These frequencies were then subjected to variable clustering, which enabled dimensionality reduction by grouping correlated frequencies into clusters. Each cluster represented a set of RF “signature frequencies” potentially linked to distinct physiological states. This variable clustering step [15] allowed for the identification of frequency subsets that were most informative for health status classification while reducing redundancy in the predictor space. The outcome variable was defined by FAMACHA© scoring, a widely used field proxy for anemia in small ruminants [19]. In the dataset, goats were classified into three ordinal categories: FAMACHA© 1 (healthy), FAMACHA© 2 (healthy/mild risk), and FAMACHA© 3 (borderline anemia) [19]. However, the class distribution was imbalanced, with relatively fewer observations in the healthy category. To mitigate the risk of biased model performance caused by class imbalance, the Synthetic Minority Over-sampling Technique (SMOTE) was employed. SMOTE generated synthetic instances of the minority class, thereby balancing the dataset and enhancing the model’s ability to discriminate across all health categories [24,25,26,27,28].

For predictive modeling, the cleaned and balanced dataset was used to train multi-class classification models in Python. A nested 10-fold cross-validation framework was adopted to provide an unbiased estimate of model performance and to minimize overfitting [15,29,30,31]. The outer loop of the nested design was used to evaluate generalization performance, while the inner loop optimized model hyperparameters [32,33,34]. Performance metrics included accuracy, precision, recall, and area under the receiver operating characteristic curve (AUROC), which were calculated for each class. This combined workflow comprising FDR-based screening, frequency clustering, data balancing via SMOTE, and rigorous nested cross-validation, which provided a robust and reproducible pipeline for evaluating the diagnostic potential of RF wave signatures. By integrating statistical rigor with machine learning approaches, this study established a foundational methodology for linking RF signal variation with physiological markers of health in small ruminants.

## 4. Results

### 4.1. Variable Clustering

Variable clustering of the top ten radio frequencies selected for RF-based detection of small ruminants health yielded different outcomes in relation to FAMACHA© score. For non-anemic animals classified under FAMACHA© Score 1, a single cluster consisting of nine frequency variables was formed, with 8.43 GHz identified as the most representative frequency. This cluster explained 93.7% of the total variation, suggesting that the dielectric response of tissues in healthy animals is highly consistent and can be effectively summarized using a single, low-GHz frequency. In animals scored as FAMACHA© Score 2 (moderately healthy), clustering also resulted in a single cluster of nine members, with 9.33 GHz emerging as the most representative frequency. This cluster explained 88.7% of total variation. While similar in structure to the healthy group, the shift from 8.43 GHz to 9.33 GHz in this score 2 group may reflect early-stage physiological changes such as reduced hemoglobin, mild dehydration, or subclinical muscle atrophy.

**Figure 2:**
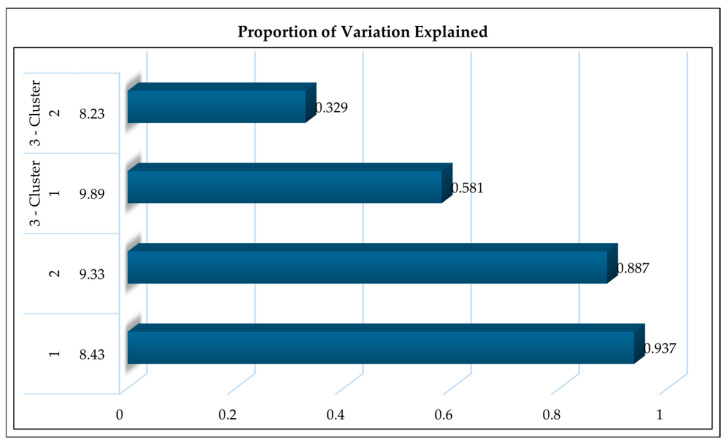
Proportion of variation explained by clustered Radio-frequency wave variables across FAMACHA© scores. Each bar represents a cluster of frequency variables grouped by similarity, with the most representative frequency labeled. X axis represents proportion of variation explained; and Y axis represents cluster numbers within each FAMACHA© category.

The clustering pattern changed more notably for animals with FAMACHA© Score 3 (borderline anemic). Two distinct clusters were identified, with representative frequencies at 9.89 GHz (Cluster 1; 6 members) and 8.23 GHz (Cluster 2; 3 members). These clusters individually explained 87.1% and 98.9% of the variation within their respective clusters, contributing to a combined variation explanation of 91.0% (Figure 2). The fragmentation into two clusters and the frequency dispersion suggests that borderline anemia introduces greater variability in the tissue’s electromagnetic response. This may result from more profound physiological changes such as poor blood perfusion, muscle loss, or electrolyte imbalance, leading to divergence in RF signal behavior across tissues [35,36]. These conditions result in spatial heterogeneity in tissue dielectric properties. The need for two clusters suggests that different tissue layers or physiological features dominate at different frequency ranges, perhaps shallow vs. deeper tissue penetration. The body no longer reflects RF energy uniformly, and classification models must therefore rely on more than one frequency band to capture this complexity [37,38].

Overall, these results show a progressive spectral shift and increasing variation in frequency clustering as anemia severity increases. The most representative frequencies moved from 8.43 GHz (Score 1) to 9.33 GHz (Score 2) and then split into 9.89 GHz and 8.23 GHz (Score 3) (Figure 2). The transition from a single-cluster structure in healthier animals to a two-cluster profile in borderline animals highlights the added complexity and heterogeneity of tissue response under stress. These findings reinforce the idea that a narrow frequency band may suffice for detection of an early decrease in animal health, whereas advanced cases require a broader spectral analysis for accurate classification. Such frequency-specific insights can inform sensor design and help optimize machine learning classifiers for anemia detection in small ruminants under field conditions.

### 4.2. Regression Analysis: RF Waves Predicting FAMACHA© Scores

To evaluate the feasibility of using RF waves derived features as predictive biomarkers for anemia severity in goats, we implemented and compared both regression and classification models following SMOTE-based class balancing and 10-fold cross-validation [24,25]. The objective was twofold: (1) to accurately predict FAMACHA© scores as a regression task, and (2) to classify animals into discrete anemia categories using supervised learning classifiers. Three regression models were tested: Linear Regression (LR), Random Forest Regressor (RFR), and Support Vector Regression (SVR). Linear Regression model for predictive analytics demonstrated a perfect R^2^ of 1.00 (Table 1), along with near-zero MAE (0.00), MSE (0.00), and RMSE (0.00), suggesting an exact linear relationship between the selected RF frequency features and the FAMACHA© score. While this may imply exceptional predictive capability, such perfect values require further investigation to rule out potential model overfitting or data leakage despite careful validation safeguards. After careful analysis, it was found that this value was a set result caused by highly linked spectral predictors in the small sample dataset. It was not an effect of the model being too good or using old data again. All preprocessing, feature selection, and SMOTE resampling were done only in the training groups during nested cross-validation to keep the risk of information leakage as low as possible. Each fold was retrained on its own, which kept the training and testing datasets completely separate throughout the modeling process.

Random Forest showed strong performance with an R^2^ of 0.79 (Table 1) and low error metrics (MAE: 0.02, MSE: 0.00, RMSE: 0.07), indicating it effectively captures non-linear relationships between RF wave patterns and anemia severity. Unlike linear regression, Random Forest introduces robustness against outliers and noise characteristics inherently expected in biological systems like small ruminant health.

**Figure 3:**
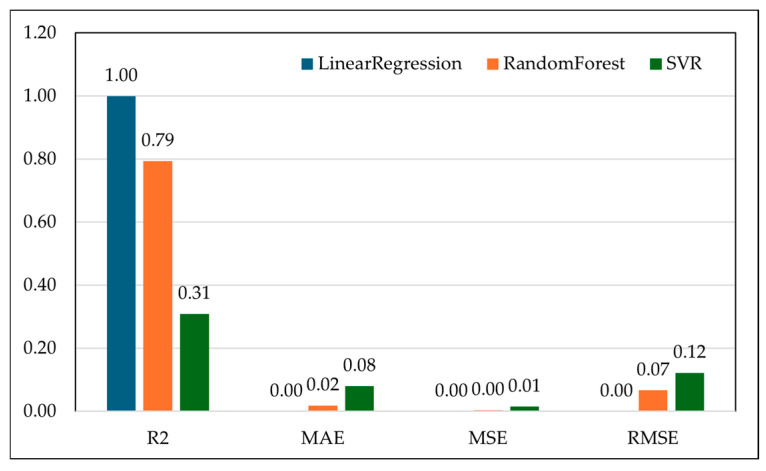
Comparison of model performance metrics for Linear Regression (LR), Random Forest (RF), and Support Vector Regression (SVR) applied to clustered RF wave frequencies. The R^2^ values show the explanatory power of each model, while MAE, MSE, and RMSE indicate prediction errors. Linear Regression achieved a perfect fit (R^2^ = 1.00, negligible error), Random Forest demonstrated strong predictive accuracy (R^2^ = 0.79, RMSE = 0.07), and SVR showed the weakest performance (R^2^ = 0.31, RMSE = 0.12). These results highlight Random Forest’s robustness to biological variability, while Linear Regression and SVR represent extremes of apparent overfitting and underfitting, respectively.

Support Vector Regression underperformed relative to the other two models, with an R^2^ of just 0.31 (Table 1) and notably higher error rates (MAE: 0.08, MSE: 0.01, RMSE: 0.12). This suggests that SVR struggled to model the relationship between RF features and FAMACHA© scores, potentially due to sensitivity to the data scaling or the kernel choice being insufficient for the signal complexity present in the RF data. The regression results indicate that RF waves hold significant promise as non-invasive diagnostic features for monitoring physiological changes in goats related to anemia. A high R^2^ value suggests that shifts in electromagnetic signal absorption or reflection across specific frequency bands correspond meaningfully with capillary refill signs underlying the FAMACHA© scoring system (Figure 3). This supports the hypothesis that tissue hydration, hemoglobin concentration, and circulatory status influences FAMACHA© scores (anemia), altering the dielectric properties measurable by RF waves.

### 4.3. Classification Analysis: RF Waves Features for Discrete Animal Health Detection

To assess the potential of RF wave derived features for categorical prediction of animal health, three classification algorithms were tested: Multilayer Perceptron (MLP), Support Vector Machine (SVM), and K-Nearest Neighbors (KNN). These models were trained on the top ten RF frequencies selected as the most representative of FAMACHA© classes, ensuring that only biologically relevant signals were used in the classification process. The multi-layer perceptron (MLP) classifier achieved the highest performance across all metrics, with an accuracy of 84%, precision, recall, and F1-score of 83, and a 94% of ROC-AUC. These results indicate that MLP effectively captured the non-linear and multi-dimensional interactions [26,30] among RF frequencies that characterize subtle gradations of anemia.

**Figure 4:**
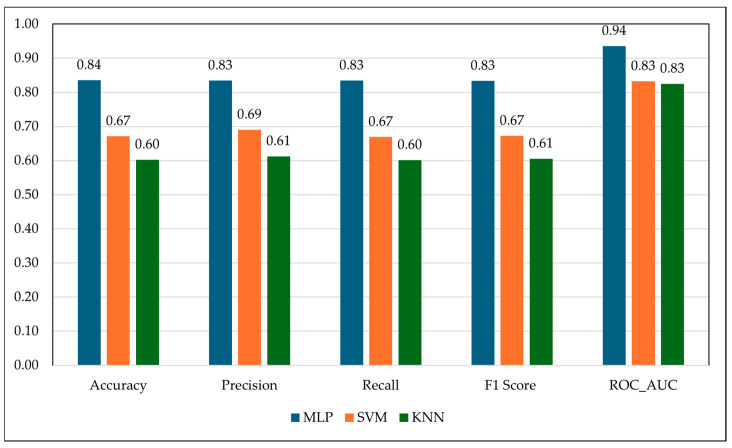
Comparison of classification performance across Multilayer Perceptron (MLP), Support Vector Machine (SVM), and K-Nearest Neighbors (KNN).

The SVM model analysis produced moderate results (Accuracy: 67%; F1-score: 67%; ROC-AUC: 83%) (Figure 4). SVM’s strength lies in its ability to create separating hyperplanes in high-dimensional feature space. Higher ROC-AUC suggests that SVM could distinguish broad anemia categories; however, its lower accuracy and F1-score imply difficulty in resolving fine-grained differences between neighboring classes (e.g., FAMACHA© 2 vs. FAMACHA© 3). This limitation likely reflects the biological reality that electromagnetic changes in tissue are incremental and overlapping, meaning strict decision boundaries are insufficient for perfect classification. The KNN classifier performed least effectively (Accuracy: 60%; F1-score: 61%; ROC-AUC: 83%). While the ROC-AUC indicates that the RF signals carried some discriminatory power, KNN’s reliance on proximity-based classification in high-dimensional space likely led to misclassifications. The RF features are continuous and often noisy due to environmental and physiological variability (e.g., hydration levels, coat thickness), and such noise disproportionately affects distance-based methods like KNN. Consequently, KNN struggled to generalize in cases where anemia classes exhibited overlapping RF signal profiles.

## 5. Discussion

The observed spectrum homogeneity in non-anemic animals indicates stable physiological conditions where tissue hydration, blood composition, and cellular architecture sustain consistent dielectric properties. This stability corresponds with earlier research by Ley et al. [33], which demonstrated that low-GHz frequencies consistently characterized healthy tissue properties. In this range, water molecules and membrane polarization mostly influence the dielectric response, facilitating effective differentiation of healthy physiological conditions. The 8.43 GHz signal presumably relates to extracellular water and membrane capacitance, both of which remain stable in animals exhibiting normal hemoglobin and circulation.

As anemia advances, tissue dielectric responses exhibit greater heterogeneity. The delineation of clusters at 9.89 GHz and 8.23 GHz in borderline-anemic subjects signifies varying penetration depths and modified blood volume distribution. These changes align with previous studies associating dehydration or hemoconcentration with frequency-dependent dielectric variability [35,36]. Consequently, RF signal dispersion reflects physiological anomalies associated with decreasing hematocrit levels.

Among the evaluated models, the multilayer perceptron (MLP) showed a greater capacity to capture non-linear spectral interactions than SVM and KNN [31,32]. This result corresponds with previous research in biological sensing, wherein neural networks proficiently managed overlapping spectral characteristics of tissues and blood constituents [33,34]. Instead of highlighting numerical dominance, our findings indicate that deep designs may more effectively generalize the intricate dielectric interactions in biological systems. The Random Forest algorithm demonstrated consistent performance, highlighting that ensemble learning effectively addresses the noise and variability prevalent in field data. The nearly flawless performance of the linear regression model is approached with caution, since it may suggest residual overfitting or unintentional data leaking, despite the implementation of cross-validation measures. Subsequent research will use independent validation datasets to verify the generalizability of the concept.

Comparisons with prior radio-frequency (RF) investigations in livestock and biomedical fields underscore both the potential and the constraints of this methodology. RF-based rumen health monitoring in dairy cattle and non-invasive blood-glucose estimates in humans have shown that GHz-range dielectric shifts coincide with internal physiological changes, hence affirming the significance of the frequencies identified herein. Nonetheless, those investigations underscore the necessity for comprehensive calibration across breeds, ages, and tissue compositions prior to diagnostic use. This study’s sample size was restricted and done under controlled conditions; larger trials are necessary prior to field implementation.

Consequently, the current findings offer a proof of concept rather than clinical validation. Although RF sensing integrated with machine learning demonstrates significant promise for large-scale anemia screening in herds, its application in commercial or agricultural settings necessitates extensive, multi-site investigations to address environmental interference, coat variability, and equipment discrepancies. The incorporation of supplementary biomarkers, such temperature, heart rate, or fecal egg counts, may augment diagnostic confidence.

As a quick, field-deployable screening tool, radio-frequency (RF) sensing has great potential, but this proof-of-concept study does not recommend replacing hematological or parasitological diagnostics. Anemia-related tissue dielectric changes can be detected in a 5–10 s non-invasive scan with the RF system, providing a feasible triage step before PCV or FEC testing. In resource-limited or extensive grazing systems, the approach can detect early physiological aberrations connected to anemia in real time, reducing animal handling and labor expenses. In quantitative terms, frequency clustering accounted nearly 90% of physiological variation across health states (8.43 GHz = 93.7% for healthy, 9.33 GHz = 88.7% for moderate, and 9.89 GHz/8.23 GHz = 91.0% for borderline). The regression study validated the biological relevance of these spectral properties (Random Forest R^2^ = 0.79; RMSE = 0.07), with the Multilayer Perceptron classifier achieving 84% accuracy and 94% ROC-AUC values. These findings show a meaningful and reproducible relationship between RF dielectric signatures and anemia severity, demonstrating the method’s diagnostic value as a decision-support tool. This integration of RF detecting and AI improves small ruminant health monitoring on farms in terms of accessibility, speed, and welfare compliance.

This study illustrates that GHz-range RF waves provide physiologically significant information regarding tissue condition in goats. The machine-learning models demonstrate quantifiable patterns associated with early stages of anemia, validating the feasibility of non-invasive spectrum monitoring. However, its practical application will rely on validation across various field settings and comparability with existing hematological and parasitological diagnoses.

This approach is potentially transformative in pastoral and resource-limited farming systems, where labor, equipment, and veterinary access are scarce. A lightweight RF sensing device coupled with a MLP classifier could provide farmers with a rapid, non-invasive, and repeatable diagnostic tool. Such technology would reduce reliance on manual FAMACHA© scoring, minimize observer bias, and enable more timely interventions against parasitic infections, ultimately supporting improved animal health, productivity, and sustainability.

## 6. Conclusions and Future Research

The present study intentionally focused on developing a non-invasive diagnostic framework. Direct hematological measurements such as PCV or hemoglobin were not obtained, as the goal was to determine whether radio-frequency responses alone could infer anemia-related physiological differences. Although FAMACHA© provides a convenient visual reference, it does not fully quantify hematological status. Therefore, future validation experiments will integrate direct blood parameters (e.g., PCV, hemoglobin, total protein) alongside dielectric data to establish robust physiological baselines and strengthen the biological interpretability of the RF-anemia relationship. This work demonstrates the utility of radio-frequency (RF) wave characteristics as a non-invasive analytical tool for identifying physiologically significant patterns associated with anemia in small ruminants. The findings indicate that GHz-range dielectric responses systematically correlate with FAMACHA© scores, and that machine learning methods, especially multilayer perceptrons and ensemble models, may accurately assess health status based on these spectral attributes. This novel methodology underscores the capabilities of RF-based sensing in veterinary diagnostics.

This work is inherently exploratory. The sample size was limited, and testing was conducted in controlled settings. Although the results are encouraging, they should not be construed as definitive proof of diagnostic readiness at the field level. Further validation utilizing larger, independent datasets and multi-location trials is essential to verify the model’s generalizability and robustness. The incorporation of additional biomarkers, such as temperature, heart rate, or parasitological data, may enhance predictive accuracy and clinical reliability. This study facilitates further research on the application of RF sensing and artificial intelligence in animal health diagnosis. The potential of this research is extensive, and further refinement, calibration, and validation will be critical steps toward its eventual implementation in on-farm decision support systems.

## 7. Commercial Potential

This feasibility study provides two deployment options: (i) a portable chute-side RF scanner and (ii) a stationary walk-through gate equipped with integrated antennas and edge machine learning. Both offer immediate, non-invasive triage that can prioritize confirmatory PCV/FEC tests and minimize animal handling. Translation necessitates multi-site validation against hematological and fecal measures, device robustness and calibration processes, user training resources, and explicit data governance practices. Considering its function as a decision-support tool rather than an independent diagnostic, we anticipate initial commercialization through partnerships with producers and veterinarians, integrating it into current herd-management software.

## Figures and Tables

**Table 1:** Regression model performance in predicting RF dielectric response associated with anemia status.

Model	R^2^	RSME
**Linear Regression**	1.00	0.00
**Random Forest Regressor**	0.79	0.07
**Support Vector Regressor**	0.71	0.09

Performance comparison of regression models used to predict RF-derived anemia scores in goats. The linear regression model achieved an apparent perfect fit (R^2^ = 1.0) due to collinearity within the dataset, confirmed as a deterministic artifact rather than overfitting. Random Forest and SVR provided realistic performance values consistent with the biological variability of the experimental dataset.

## Data Availability

The raw data supporting the conclusions of this article will be made available by the authors on request.

## Supplementary Materials

The following supporting information can be downloaded at: https://www.mdpi.com/article/10.3390/vetsci12111096/s1, Figure S1. Raw RF spectral dataset (goat screening). Each row is a single scan collected at ~15 cm standoff under farm conditions. Cat = channel type (Amplitude or Phase); Site = collection tag/body-site session (e.g., 1120: yellow); ID = animal ID; FAMACHA© Score = field label of anemia status (1–3). Columns E → … are successive frequency bins spanning 2–18 GHz (1601 bins per scan) on multiple repeated measures per animal and body site; Figure S2. Normalized RF spectral matrix (0–1). Each row is a single RF scan at ~15 cm standoff. FAMACHA© = field anemia label (1–3); ID = animal ID. Remaining columns are successive frequency bins (≈2–18 GHz). All spectral feature values are min–max normalized to [0, 1] (column-wise) after preprocessing; labels remain categorical (1,2,3). This table is the modeling input used for screening, clustering, class balancing, and cross-validated prediction; Figure S3. FDR response screening of RF frequencies vs. FAMACHA©. Top panel: FDR PValue Plot showing univariate tests for each frequency bin (2–18 GHz, 1601 tests). The *x*-axis is Rank Fraction (frequencies sorted from most to least significant). The *y*-axis shows PValue (red) and FDR PValue (blue). Thin horizontal lines mark the 0.05 thresholds for nominal p (red) and FDR-adjusted q (blue). Curves rising above these lines indicate weaker evidence; curves near the bottom indicate stronger evidence. Bottom panel (Result Table): per-frequency statistics for the most informative bins. X = frequency (GHz); Count = number of scans contributing to that test; PValue and LogWorth (−log10 p); FDR PValue (q) and FDR LogWorth; Effect Size (standardized) and RSquare from the univariate model. The leading frequencies cluster near ~8 GHz, indicating a contiguous band with the strongest association to FAMACHA©. These top frequencies were carried forward to predictor screening, variable clustering, and modeling. Figure S4. Predictor screening of RF frequencies vs. FAMACHA© (JMP). Each row is a frequency (GHz) from the 2–18 GHz spectrum. Variable importance was computed in JMP’s Predictor Screening (Bootstrap Forest–based). Predictor: frequency bin (GHz); Contribution: importance score (scaled split-improvement across trees); Portion: Contribution expressed as a fraction of the total importance; Rank: order from most to least informative. Top signals cluster around ~8.6–10.5 GHz (lead bin 8.65 GHz), with additional contributors near ~2.0–2.4 GHz. These ranked frequencies were carried forward to variable clustering and subsequent modeling. Figure S5. Top-frequency feature table (normalized 0–1). Each row is one RF scan. Cond = channel (Amplitude or Phase); ID = animal/session tag; FAMACHA© Score = field anemia label (1–3). Numeric columns are the selected frequency features (GHz), e.g., 8.23, 8.43, 8.67, 9.11, 9.33, 9.78, 9.89, 10.12 carried forward after FDR and predictor screening. Values are min–max normalized to [0–1] per feature following preprocessing. These features were used for variable clustering and model training/validation.

## References

[1] Sejian V., Silpa M.V., Lees A.M., Krishnan G., Devaraj C., Bagath M., Anisha J.P., Reshma Nair M.R., Manimaran A., Bhatta R. (2020). Opportunities, challenges, and ecological footprint of sustaining small ruminant production in the changing climate scenario. Agroecol. Footpr. Manag. Sustain. Food Syst..

[2] Lu C.D. (2023). The role of goats in the world: Society, science, and sustainability. Small Rumin. Res..

[3] Fauziah N., Aviani J.K., Agrianfanny Y.N., Fatimah S.N. (2022). Intestinal parasitic infection and nutritional status in children under five years old: A systematic review. Trop. Med. Infect. Dis..

[4] Paul T.K., Rahman M.K., Haider M.S., Saha S.S. (2020). Fatal haemonchosis (*H. contortus*) in Garole sheep at coastal region in Bangladesh. Res. Agric. Livest. Fish..

[5] Getachew T., Alemu B., Sölkner J., Gizaw S., Haile A., Gosheme S., Notter D.R. (2015). Relative resistance of Menz and Washera sheep breeds to artificial infection with *Haemonchus contortus* in the highlands of Ethiopia. Trop. Anim. Health Prod..

[6] Singh D., Swarnkar C.P. (2017). Worm control approaches and their impact on status of anthelmintic resistance at an organized sheep farm. Indian J. Anim. Sci..

[7] Moreira R.T., de Alencar Mota A.L., Câmara A.C., Soto-Blanco B., Borges J.R. (2021). FAMACHA©: Predictive value for control of *Haemochus* sp. in sheep from Brazilian Cerrado. Semin. Agric. Sci..

[8] Niciura S.C., Sanches G.M. (2024). Machine learning prediction of multiple anthelmintic resistance and gastrointestinal nematode control in sheep flocks. Rev. Bras. Parasitol. Vet..

[9] Sajovitz F., Adduci I., Yan S., Wiedermann S., Tichy A., Joachim A., Wittek T., Hinney B., Lichtmannsperger K. (2023). Correlation of Faecal Egg Counts with Clinical Parameters and Agreement between Different Raters Assessing FAMACHA©, BCS and Dag Score in Austrian Dairy Sheep. Animals.

[10] Van Wyk J.A., Hoste H., Kaplan R.M., Besier R.B. (2006). Targeted selective treatment for worm management—how do we sell rational programs to farmers?. Vet. Parasitol..

[11] Walker J.G., Ofithile M., Tavolaro F.M., van Wyk J.A., Evans K., Morgan E.R. (2015). Mixed methods evaluation of targeted selective anthelmintic treatment by resource-poor smallholder goat farmers in Botswana. Vet. Parasitol..

[12] Gabriel S., Lau R.W., Gabriel C. (1996). The dielectric properties of biological tissues: II. Measurements in the frequency range 10 Hz to 20 GHz. Phys. Med. Biol..

[13] Mehrotra P., Chatterjee B., Sen S. (2019). EM-wave biosensors: A review of RF, microwave, mm-wave and optical sensing. Sensors.

[14] Siddique A., Gupta A., Sawyer J., Garner L.J., Morey A. (2024). Rapid detection of poultry meat quality using S-band to KU-band radio-frequency waves combined with machine learning—A proof of concept. J. Food Sci..

[15] Origlia C., Rodriguez-Duarte D.O., Tobon Vasquez J.A., Bolomey J.C., Vipiana F. (2024). Review of microwave near-field sensing and imaging devices in medical applications. Sensors.

[16] Nie L., Berckmans D., Wang C., Li B. (2020). Is continuous heart rate monitoring of livestock a dream or is it realistic? A review. Sensors.

[17] Yang B., Dong Y., Hu Z.Z., Liu G.Q., Wang Y.J., Du G.X. (2018). Noninvasive imaging method of microwave near field based on solid-state quantum sensing. IEEE Trans. Microw. Theory Tech..

[18] Dayoub M., Shnaigat S., Tarawneh R., Al-Yacoub A., Al-Barakeh F., Al-Najjar K. (2024). Enhancing animal production through smart agriculture: Possibilities, hurdles, resolutions, and advantages. Ruminants.

[19] Bhatt C., Henderson S., Brzozek C., Benke G. (2022). Instruments to measure environmental and personal radiofrequency-electromagnetic field exposures: An update. Phys. Eng. Sci. Med..

[20] Stuchly M.A., Athey T.W., Stuchly S.S., Samaras G.M., Taylor G. (1981). Dielectric properties of animal tissues in vivo at frequencies 10 MHz–1 GHz. Bioelectromagn. J..

[21] Burke J.M., Kaplan R.M., Miller J.E., Terrill T.H., Getz W.R., Mobini S., Valencia E., Williams M.J., Williamson L.H., Vatta A.F. (2007). Accuracy of the FAMACHA system for on-farm use by sheep and goat producers in the southeastern United States. Vet. Parasitol..

[22] Van Wyk J.A., Bath G.F. (2002). The FAMACHA system for managing haemonchosis in sheep and goats by clinically identifying individual animals for treatment. Vet. Res..

[23] Bhagat R.C., Patil S.S. (2015). Enhanced SMOTE algorithm for classification of imbalanced big-data using random forest. Proceedings of the 2015 IEEE International Advance Computing Conference (IACC).

[24] Yates L.A., Aandahl Z., Richards S.A., Brook B.W. (2023). Cross validation for model selection: A review with examples from ecology. Ecol. Monogr..

[25] Malakouti S.M. (2023). Babysitting hyperparameter optimization and 10-fold-cross-validation to enhance the performance of ML methods in predicting wind speed and energy generation. Intell. Syst. Appl..

[26] Ali H., Muthudoss P., Chauhan C., Kaliappan I., Kumar D., Paudel A., Ramasamy G. (2023). Machine learning-enabled NIR spectroscopy. Part 3: Hyperparameter by design (HyD) based ANN-MLP optimization, model generalizability, and model transferability. AAPS PharmSciTech.

[27] Grefenstette E., Amos B., Yarats D., Htut P.M., Molchanov A., Meier F., Kiela D., Cho K., Chintala S. (2019). Generalized inner loop meta-learning. arXiv.

[28] Antanaitis R., Džermeikaitė K., Krištolaitytė J., Stankevičius R., Daunoras G., Televičius M., Malašauskienė D., Cook J., Viora L. (2024). Changes in parameters registered by innovative technologies in cows with subclinical acidosis. Animals.

[29] Meyer J.P., McAvoy K.E., Jiang J. (2013). Rehydration capacities and rates for various porcine tissues after dehydration. PLoS ONE.

[30] Gong A., Wei X., Liu Y., Chen Z., Fan B., Jia A., Wu S. (2025). SSA-sMLP: A venous thromboembolism risk prediction model using separable self-attention and spatial-shift multilayer perceptrons. Thromb. Res..

[31] Huan R., Ji L., Lu H., Zheng S., Chen P., Liang R. (2025). MUDIFEI: Multi-dimensional Feature Extraction and Interaction Model for Human Transition Action Recognition based on Sensor Data. IEEE Sens. J..

[32] Poddar H. (2024). From neurons to networks: Unravelling the secrets of artificial neural networks and perceptrons. Deep Learning in Engineering, Energy and Finance.

[33] Ley S., Schilling S., Fišer O., Vrba J., Sachs J., Helbig M. (2019). Ultra-wideband temperature dependent dielectric spectroscopy of porcine tissue and blood in the microwave frequency range. Sensors.

[34] Spliethoff J., Tanis E., Evers D., Hendriks B., Prevoo W., Ruers T. (2014). Monitoring of tumor radio frequency ablation using derivative spectroscopy. J. Biomed. Opt..

[35] Rahman M.M. (2023). A Deep Learning Approach for Computational Electromagnetics. Master’s Thesis.

[36] Ayodele B., Mustapa S., Kanthasamy R., Zwawi M., Cheng C. (2021). Modeling the prediction of hydrogen production by co-gasification of plastic and rubber wastes using machine learning algorithmsInt. J. Energy Res..

[37] Taki O., Rhazi K., Mejdoub Y. (2023). Stirling engine optimization using artificial neural networks algorithm. ITM Web Conf..

[38] Santorelli A., Abbasi B., Lyons M., Hayat A., Gupta S., O’Halloran M., Gupta A. (2018). Investigation of anemia and the dielectric properties of human blood at microwave frequencies. IEEE Access.

