# Supplementary material for: Radiofrequency Wave Sensing for Rapid Animal Health Monitoring: A Proof-of-Concept Study
Source: Vet Sci. 2025 Nov 18;12(11):1096. doi: 10.3390/vetsci12111096 (PMC12656894; doi:10.3390/vetsci12111096)
Supplement: Supplementary file 1 [file vetsci-12-01096-s001.zip › vetsci-3910655-supplementary.pdf]

Radiofrequency Wave Sensing for Rapid Animal Health Monitoring: A Proof-of-Concept Study

Aftab Siddique [1,*], Ramya Kota [1], Goutham Kumar Isai [1], Davia Brown [1], Oreta Samples [1], Niki Whitley [1],
Jan van Wyk [2] and Thomas H. Terrill [1]

[1] Fort Valley State University, Fort Valley, GA 31030, USA; rkota@wildcat.fvsu.edu (R.K.); gisai@wildcat.fvsu.edu (G.K.I.); dbrow212@wildcat.fvsu.edu (D.B.); phaneendra.batchu@fvsu.edu (P.B); sampleso@fvsu.edu (O.S.); whitleyn@fvsu.edu (N.W.); terrillt@fvsu.edu (T.H.T.)
[2] Department of Veterinary Tropical Diseases, Faculty of Veterinary Science, University of Pretoria, Private Bag x04, Onderstepoort 0110, South Africa; jan.vanwyk@up.ac.za (J.v.W)
* Correspondence: aftab.siddique@fvsu.edu ; Tel.: 478-391-1080

Simple Summary

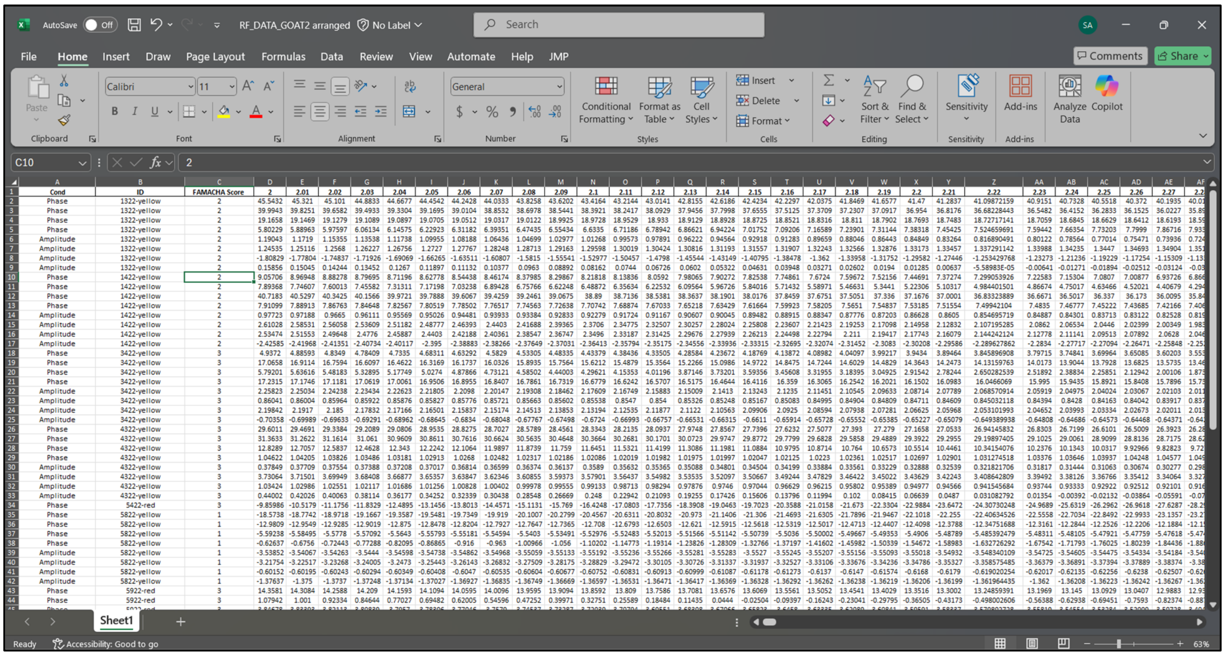

Figure S1. Raw RF spectral dataset (goat screening). Each row is a single scan collected at ~15 cm standoff under farm conditions. Cat = channel type (Amplitude or Phase); Site = collection tag/body-site session (e.g., 1120: yellow); ID = animal ID; FAMACHA Score = field label of anemia status (1–3). Columns E → … are successive frequency bins spanning 2–18 GHz (1,601 bins per scan) on multiple repeated measures per animal and body site.

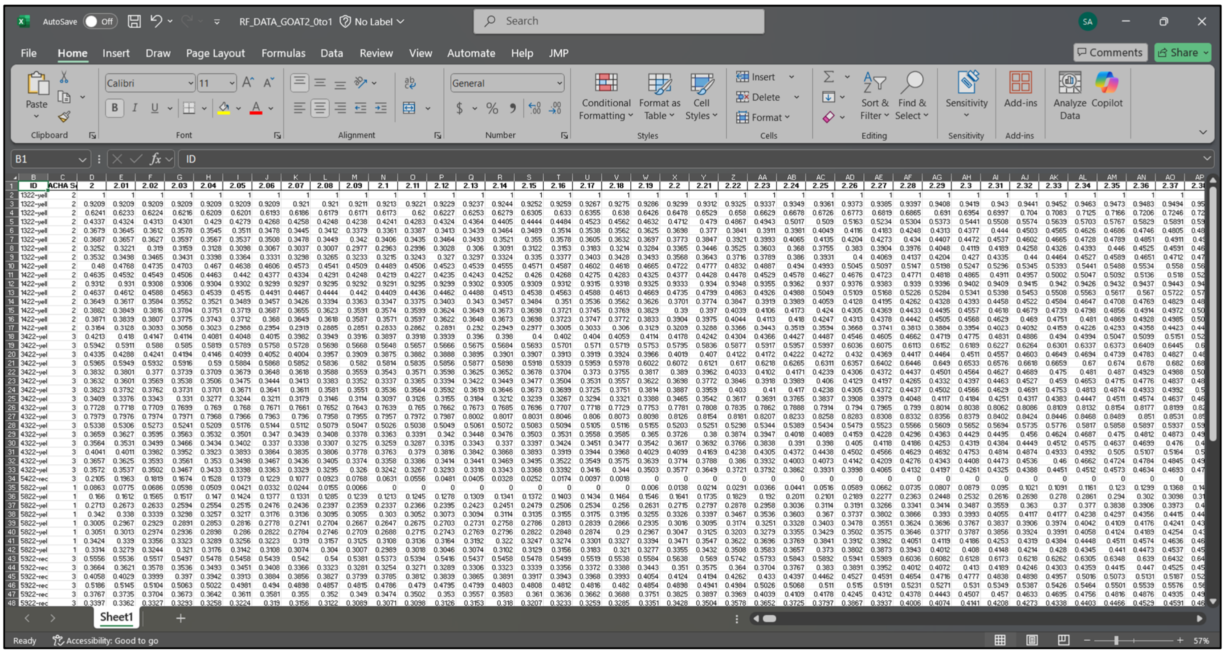

Figure S2. Normalized RF spectral matrix (0–1). Each row is a single RF scan at ~15 cm standoff. FAMACHA = field anemia label (1–3); ID = animal ID. Remaining columns are successive frequency bins (≈2–18 GHz). All spectral feature values are min–max normalized to [0,1] (column-wise) after preprocessing; labels remain categorical (1,2,3). This table is the modeling input used for screening, clustering, class balancing, and cross-validated prediction.

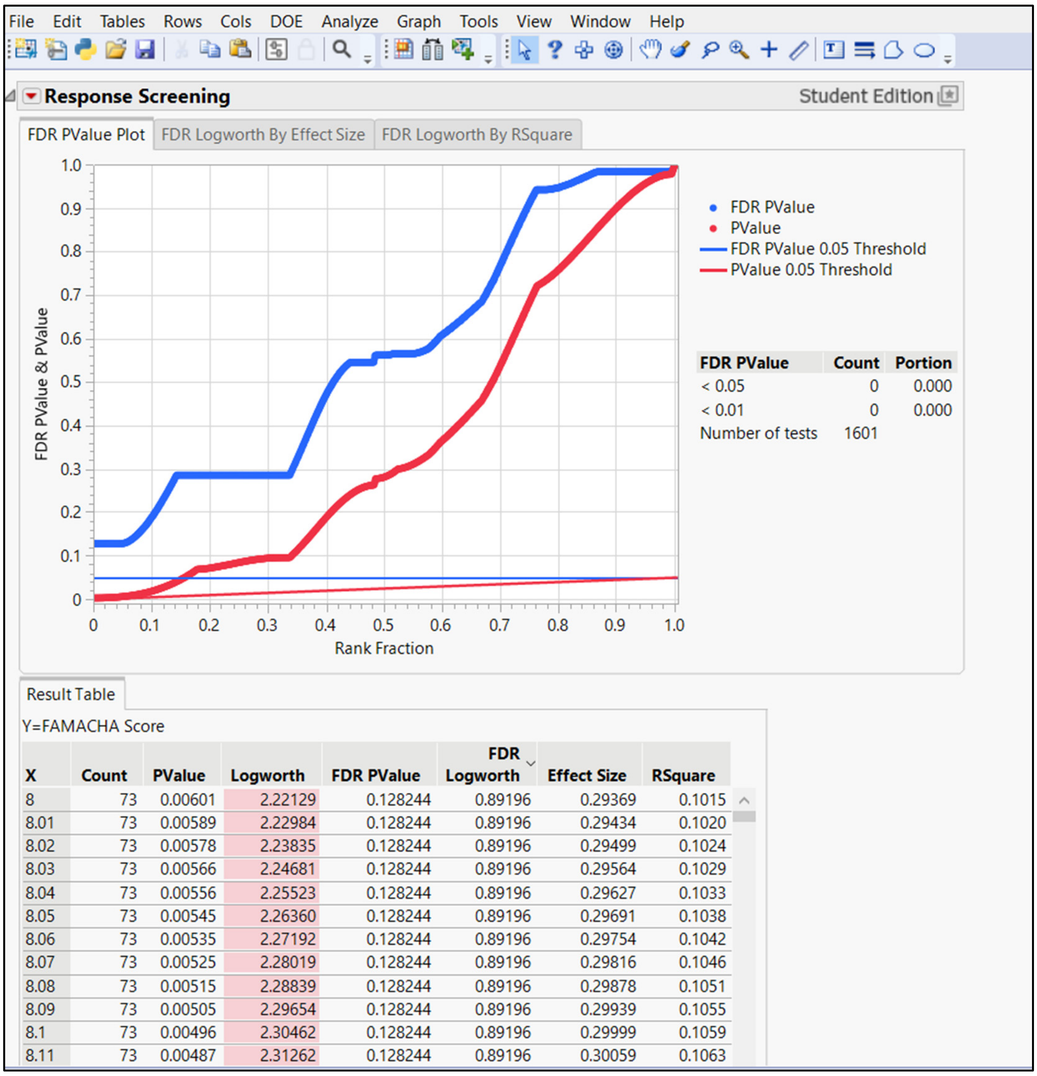

X	Count	PValue	Logworth	FDR PValue	FDR Logworth	Effect Size	RSquare
8	73	0.00601	2.22129	0.128244	0.89196	0.29369	0.1015
8.01	73	0.00589	2.22984	0.128244	0.89196	0.29434	0.1020
8.02	73	0.00578	2.23835	0.128244	0.89196	0.29499	0.1024
8.03	73	0.00566	2.24681	0.128244	0.89196	0.29564	0.1029
8.04	73	0.00556	2.25523	0.128244	0.89196	0.29627	0.1033
8.05	73	0.00545	2.26360	0.128244	0.89196	0.29691	0.1038
8.06	73	0.00535	2.27192	0.128244	0.89196	0.29754	0.1042
8.07	73	0.00525	2.28019	0.128244	0.89196	0.29816	0.1046
8.08	73	0.00515	2.28839	0.128244	0.89196	0.29878	0.1051
8.09	73	0.00505	2.29654	0.128244	0.89196	0.29939	0.1055
8.1	73	0.00496	2.30462	0.128244	0.89196	0.29999	0.1059
8.11	73	0.00487	2.31262	0.128244	0.89196	0.30059	0.1063

Figure S3. FDR response screening of RF frequencies vs. FAMACHA. Top panel: FDR PValue Plot showing univariate tests for each frequency bin (2–18 GHz, 1,601 tests). The x-axis is Rank Fraction (frequencies sorted from most to least significant). The y-axis shows PValue (red) and FDR PValue (blue). Thin horizontal lines mark the 0.05 thresholds for nominal p (red) and FDR-adjusted q (blue). Curves rising above these lines indicate weaker evidence; curves near the bottom indicate stronger evidence. Bottom panel (Result Table): per-frequency statistics for the most informative bins. X = frequency (GHz); Count = number of scans contributing to that test; PValue and LogWorth (−log10 p); FDR PValue (q) and FDR LogWorth; Effect Size (standardized) and RSquare from the univariate model. The leading frequencies cluster near ~8 GHz, indicating a contiguous band with the strongest association to FAMACHA. These top frequencies were carried forward to predictor screening, variable clustering, and modeling.

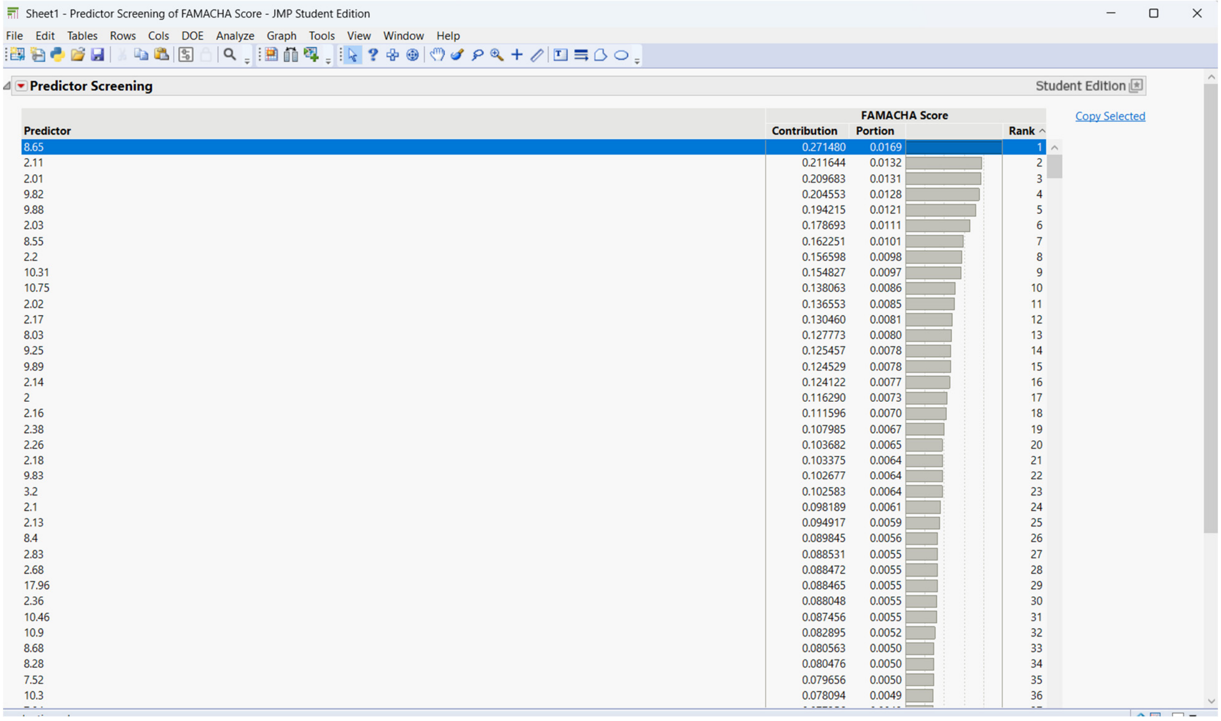

Sheet1 - Predictor Screening of FAMACHA Score - JMP Student Edition

Predictor Screening

Predictor	FAMACHA Score Contribution	Portion	Rank
8.65	0.271480	0.0169	1
2.11	0.211644	0.0132	2
2.01	0.209683	0.0131	3
9.82	0.204553	0.0128	4
9.88	0.194215	0.0121	5
2.03	0.178693	0.0111	6
8.55	0.162251	0.0101	7
2.2	0.156598	0.0098	8
10.31	0.154827	0.0097	9
10.75	0.138063	0.0086	10
2.02	0.136553	0.0085	11
2.17	0.130460	0.0081	12
8.03	0.127773	0.0080	13
9.25	0.125457	0.0078	14
9.89	0.124529	0.0078	15
2.14	0.124122	0.0077	16
2	0.116290	0.0073	17
2.16	0.111596	0.0070	18
2.38	0.107985	0.0067	19
2.26	0.103682	0.0065	20
2.18	0.103375	0.0064	21
9.83	0.102677	0.0064	22
3.2	0.102583	0.0064	23
2.1	0.098189	0.0061	24
2.13	0.094917	0.0059	25
8.4	0.089845	0.0056	26
2.83	0.088531	0.0055	27
2.68	0.088472	0.0055	28
17.96	0.088465	0.0055	29
2.36	0.088048	0.0055	30
10.46	0.087456	0.0055	31
10.9	0.082895	0.0052	32
8.68	0.080563	0.0050	33
8.28	0.080476	0.0050	34
7.52	0.079656	0.0050	35
10.3	0.078094	0.0049	36

Figure S4. Predictor screening of RF frequencies vs. FAMACHA (JMP). Each row is a frequency (GHz) from the 2–18 GHz spectrum. Variable importance was computed in JMP's Predictor Screening (Bootstrap Forest–based). Predictor: frequency bin (GHz); Contribution: importance score (scaled split-improvement across trees); Portion: Contribution expressed as a fraction of the total importance; Rank: order from most to least informative. Top signals cluster around ~8.6–10.5 GHz (lead bin 8.65 GHz), with additional contributors near ~2.0–2.4 GHz. These ranked frequencies were carried forward to variable clustering and subsequent modeling.

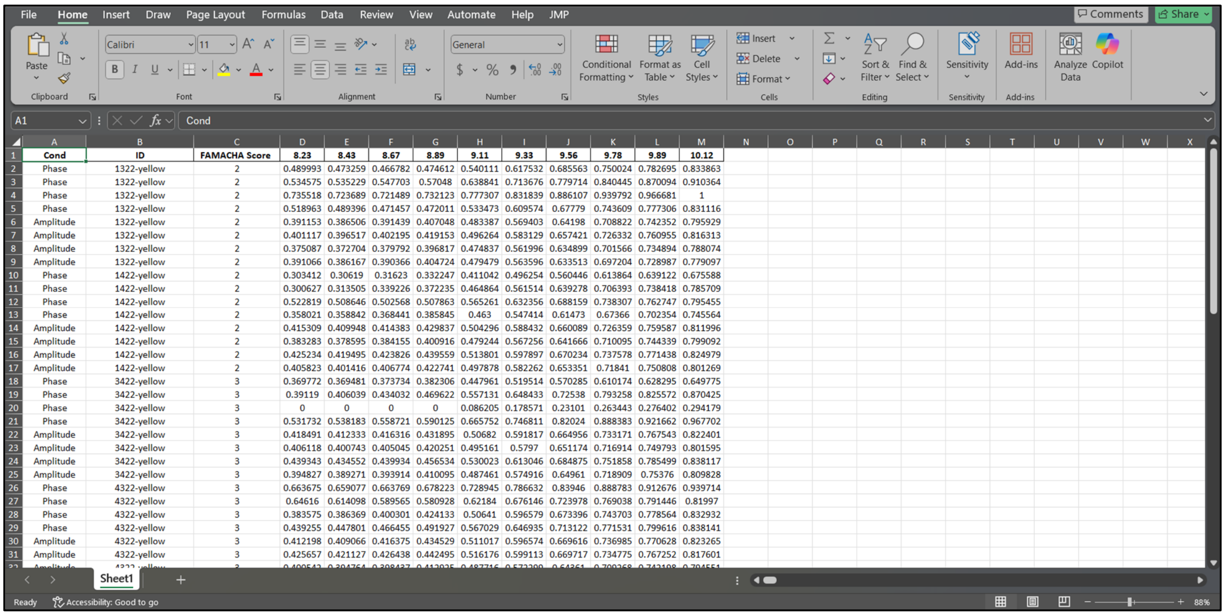

Cond	ID	FAMACHA Score	8.23	8.43	8.67	8.89	9.11	9.33	9.56	9.78	9.89	10.12
Phase	1322-yellow	2	0.489993	0.473259	0.466782	0.474612	0.540111	0.617532	0.685563	0.750024	0.782695	0.833863
Phase	1322-yellow	2	0.534575	0.535229	0.547703	0.57048	0.638841	0.713676	0.779714	0.840445	0.870094	0.910364
Phase	1322-yellow	2	0.735518	0.723689	0.721489	0.732123	0.777307	0.831839	0.886107	0.939792	0.966681	1
Phase	1322-yellow	2	0.518963	0.489396	0.471457	0.472011	0.533473	0.609574	0.67779	0.743609	0.777306	0.831116
Amplitude	1322-yellow	2	0.391153	0.386506	0.391439	0.407048	0.483387	0.569403	0.64198	0.708822	0.742352	0.795929
Amplitude	1322-yellow	2	0.401117	0.396517	0.402195	0.419153	0.496264	0.583129	0.657421	0.726332	0.760955	0.816313
Amplitude	1322-yellow	2	0.375087	0.372704	0.379792	0.396817	0.474837	0.561996	0.634899	0.701566	0.734894	0.788074
Amplitude	1322-yellow	2	0.391066	0.386167	0.390366	0.404724	0.479479	0.563596	0.633513	0.697204	0.728987	0.779097
Phase	1422-yellow	2	0.303412	0.30619	0.31623	0.332247	0.411042	0.496254	0.560446	0.613864	0.639122	0.675588
Phase	1422-yellow	2	0.300627	0.313505	0.339226	0.372235	0.464864	0.561514	0.639278	0.706393	0.738418	0.785709
Phase	1422-yellow	2	0.522819	0.508646	0.502568	0.507863	0.565261	0.632356	0.688159	0.738307	0.762747	0.795455
Phase	1422-yellow	2	0.358021	0.358842	0.368441	0.385845	0.463	0.547414	0.61473	0.67366	0.702354	0.745564
Amplitude	1422-yellow	2	0.415309	0.409948	0.414383	0.429837	0.504296	0.588432	0.660089	0.726359	0.759587	0.811996
Amplitude	1422-yellow	2	0.383283	0.378595	0.384155	0.400916	0.479244	0.567256	0.641666	0.710095	0.744339	0.799092
Amplitude	1422-yellow	2	0.425234	0.419495	0.423826	0.439559	0.513801	0.597897	0.670234	0.737578	0.771438	0.824979
Amplitude	1422-yellow	2	0.405823	0.401416	0.406774	0.422741	0.497878	0.582262	0.653351	0.71841	0.750808	0.801269
Phase	3422-yellow	3	0.369772	0.369481	0.373734	0.382306	0.447961	0.519514	0.570285	0.610174	0.628295	0.649775
Phase	3422-yellow	3	0.39119	0.406039	0.434032	0.469622	0.557131	0.648433	0.72538	0.793258	0.825572	0.870425
Phase	3422-yellow	3	0	0	0	0	0.086205	0.178571	0.23101	0.263443	0.276402	0.294179
Phase	3422-yellow	3	0.531732	0.538183	0.558721	0.590125	0.665752	0.746811	0.82024	0.888383	0.921662	0.967702
Amplitude	3422-yellow	3	0.418491	0.412333	0.416316	0.431895	0.50682	0.591817	0.664956	0.733171	0.767543	0.822401
Amplitude	3422-yellow	3	0.406118	0.400743	0.405045	0.420251	0.495161	0.5797	0.651174	0.716914	0.749793	0.801595
Amplitude	3422-yellow	3	0.439343	0.434552	0.439934	0.456534	0.530023	0.613046	0.684875	0.751858	0.785499	0.838117
Amplitude	3422-yellow	3	0.394827	0.389271	0.393914	0.410095	0.487461	0.574916	0.64961	0.718909	0.75376	0.809828
Phase	4322-yellow	3	0.663675	0.659077	0.663769	0.678223	0.728945	0.786632	0.83946	0.888783	0.912676	0.939714
Phase	4322-yellow	3	0.64616	0.614098	0.589565	0.580928	0.62184	0.676146	0.723978	0.769038	0.791446	0.81997
Phase	4322-yellow	3	0.383575	0.386369	0.400301	0.424133	0.50641	0.596579	0.673396	0.743703	0.778564	0.832932
Phase	4322-yellow	3	0.439255	0.447801	0.466455	0.491927	0.567029	0.646935	0.713122	0.771531	0.799616	0.838141
Amplitude	4322-yellow	3	0.412198	0.409066	0.416375	0.434529	0.511017	0.596574	0.669616	0.736985	0.770628	0.823265
Amplitude	4322-yellow	3	0.425657	0.421127	0.426438	0.442495	0.516176	0.599113	0.669717	0.734775	0.767252	0.817601
[illegible]	[illegible]	[illegible]	[illegible]	[illegible]	[illegible]	[illegible]	[illegible]	[illegible]	[illegible]	[illegible]	[illegible]	[illegible]

Figure S5. Top-frequency feature table (normalized 0–1). Each row is one RF scan. Cond = channel (Amplitude or Phase); ID = animal/session tag; FAMACHA Score = field anemia label (1–3). Numeric columns are the selected frequency features (GHz) e.g., 8.23, 8.43, 8.67, 9.11, 9.33, 9.78, 9.89, 10.12—carried forward after FDR and predictor screening. Values are min–max normalized to [0–1] per feature following preprocessing. These features were used for variable clustering and model training/validation.
